# Estimation of nitric oxide as an inflammatory marker in periodontitis

**DOI:** 10.4103/0972-124X.55842

**Published:** 2009

**Authors:** K. B. Menaka, Amitha Ramesh, Biju Thomas, N. Suchetha Kumari

**Affiliations:** *Department of Periodontics, A. B. Shetty Memorial Institute of Dental Sciences, Mangalore, Karnataka, India*; 1 *Department of Biochemistry, K.S. Hegde Medical Academy, Mangalore, Karnataka, India*

**Keywords:** Inflammatory marker, nitric oxide, periodontitis

## Abstract

Nitric oxide (NO) is not only important in host defense and homeostasis but it is also regarded as harmful and has been implicated in the pathogenesis of a wide variety of inflammatory and autoimmune diseases. The presence of NO in periodontal disease may reflect the participation of an additional mediator of bone resorption responsible for disease progression. The aim of this study was to assess the level of NO in serum in chronic periodontitis, and correlate these levels with the severity of periodontal disease. Sixty subjects participated in the study and were divided into two groups. NO levels were assayed by measuring the accumulation of stable oxidative metabolite, nitrite with Griess reaction. Results showed subjects with periodontitis had significantly high nitrite in serum than healthy subjects. NO production is increased in periodontal disease, this will enable us to understand its role in disease progression and selective inhibition of NO may be of therapeutic utility in limiting the progression of periodontitis.

## INTRODUCTION

Nitric oxide (NO) is a ubiquitous intercellular messenger molecule with important cardiovascular, neurological, and immune functions. NO is a short-lived, reactive free radical that participates in a variety of reactions. NO-mediated activation of soluble guanylate cyclase is responsible for signal transduction and for most of its physiological roles;[1] however, excess of NO can exert cytotoxic effects.[2] This may involve both i) direct toxicity, *e.g*., the reaction of NO with iron-containing enzymes of the respiratory cycle and of the DNA synthetic pathway, and ii) the interaction of NO with free radicals like superoxide ion (O_2_^-^) to form peroynitrite (ONOO^-^), which is a potent oxidizing molecule capable of eliciting lipid peroxidation and cellular damage.[3]

NO is produced in mammalian cells by a group of isoenzymes collectively termed NO synthases (NOS). All forms of NOS catalyze the conversion of L-arginine to L-citrulline in an NADPH-dependent manner, producing NO from the terminal *N*-guanidino group of L-arginine.[4] NOS exists as three distinct isoforms, namely, endothelial NOS (eNOS), neural NOS (bNOS), and inducible NOS (iNOS).[5] eNOS and bNOS are constitutive (cNOS) and release small amounts of NO for short periods following receptor stimulation. In contrast, iNOS is expressed in response to proinflammatory stimuli and produces large amounts of NO for sustained time periods. NO produced in high concentrations proves to be crucial in nonspecific host defence, is cytotoxic against fungal, bacterial, and protozoal organisms as well as tumor cells. Recent data indicated that NO may be less toxic independently than it has originally been proposed, and the formation of peroxynitrite may be responsible for an important part of the NO-related cytotoxicity.[1]

A chronic inflammatory disease of the periodontal tissues is periodontitis, one of the most frequently occurring human diseases and which is frequently of bacterial origin. The toxins, enzymes, and metabolities of the bacteria (predominantly Gram-negative, anaerobic) present in the dental plaque play a key role in the initiation of the inflammatory process. It is conceivable that endotoxin(s) of the Gram-negative bacteria and/or proinflammatory cytokines produced by inflammatory cells trigger resident and/or immigrant cell populations for the expression of iNOS.[6] High levels of NO produced play a role in nonspecific immunity, and wall components of Gram-negative bacteria killed by NO or peroxynitrite may trigger a positive feedback cycle, whereby bacterial wall products induce more NO production. Although the production of NO or peroxynitrite serves to induce killing or stasis of the invading microorganisms, the excessive production of these species may also lead to cytotoxicity towards the host tissues, causing tissue breakdown via multiple mechanisms, including oxidation and nitration reactions, inhibition of energy-generating enzymes, and triggering DNA injury.[5]

Furthermore, NO may activate proinflammatory enzymes such as cyclooxygenase and metalloproteinases, which, in turn, may also contribute to periodontal tissue damage. Thus, in periodontitis, iNOS expression plays a beneficial as well as a detrimental role. Beneficial effects may include antimicrobial activity, immune modulation, inhibition of microvascular thrombosis, as well as increased tissue perfusion.[7] On the other hand, detrimental effects may include a cytotoxic action towards the host tissues, including the alveolar bone. Pro- or antiinflammatory properties may vary according to NO concentration, the potential for the formation of toxic derivatives, the site of the pathological process, and the adaptive response of the target cell. The evaluation of the involvement of nitric oxide in periodontal disease will enable us to understand the complexity of periodontal disease progression.

## MATERIALS AND METHODS

The present study was conducted on 60 subjects, irrespective of gender, in the age group of 18-45 years who attended the Department of Periodontics, A.B. Shetty Memorial Institute Of Dental Sciences, Mangalore. Systemically healthy subjects, nonsmokers, and those without any history of any antibiotic therapy up to six months before the study were selected as subjects. The present study was divided into two groups: Control group or Group 1, comprised of clinically healthy gingival tissues (*n* = 30), and Case group or Group 2, subjects of chronic periodontitis with pocket depth >5 mm and clinical attachment loss of > 4 mm (*n* = 30).

### Collection of samples

Subjects' consent was taken prior to the collection of samples. About 2 mL of venous blood was drawn from the patient's arm to estimate the serum nitric oxide levels. The blood samples were centrifuged at 3000 rpm for about 10 min to collect the serum, followed by biochemical estimation of nitric oxide.

### Estimation of nitric oxide concentration

The level of NO was estimated as nitrite, a NO metabolite, in control and case group samples, because NO is a highly reactive free radical gas that is a ready oxidizer and remains stored in tissues as Nitrates (NO_3_^-^) or Nitrite (NO_2_^-^). Thus, NO concentration can be estimated by measuring concentrations of NO_3_^-^ and NO_2_^-^ in combination. The simplest technique is the monitoring of reduction of NO_3_^-^ to NO_2_^-^ by nitrate reductase or metallic catalyst, followed by the calorimetric Griess Reaction to measure NO_2_^-^ levels (nitrite levels).[8]

Sample solutions were taken in test tubes and treated with Griess reagent (1% sulphanilamide, 0.1% naphthylethylenediamine dihydrochloride and 2.5% hydrochloric acid). The colorimetric reaction was allowed to proceed for 10 min at room temperature, and optical density was measured at 550 nm using a spectrophotometer. The concentrations of nitrite were calculated from a standard curve established with serial dilutions of sodium nitrite.


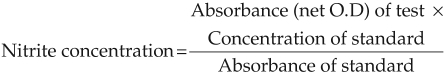


## RESULTS

**Figure 1:**
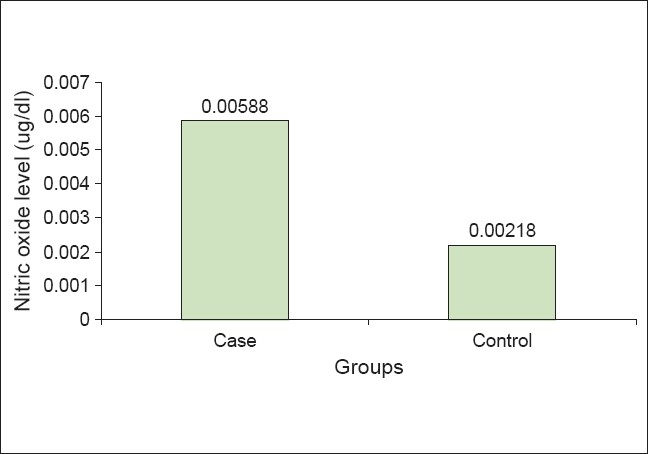
Comparison of nitric oxide level in case and controls

Table 1 shows the means and standard deviations of the nitrite levels in the case and control groups. Table 2 shows the *t*-test for equality of means. *P* < 0.05, on comparing between the two groups, clearly demonstrated the statistically significant increase in NO expression in periodontal disease. Figure 1 shows a bar graph comparing nitric oxide levels in the case and control groups.

**Table 1:** Nitrite concentration in study subjects

	Group	*N*	Mean	Std. deviation	Std. error mean
Nitric oxide	Case	30	0.005877	0.0009991	0.0001811
μg/dL	Control	30	0.002180	0.0004452	0.0000813

**Table 2:** Independent sample test

	*t*-test for equality of means				
	
	T	*P* value	Mean difference	95% confidence interval of the difference	
				
				Lower	Upper
Nitric oxide	18.623	0.000	0.0036967	0.0032993	0.0040940
μg/dL					

## DISCUSSION

Inflammation and infection are the hallmarks of periodontal disease. Most, if not all forms of human periodontal disease, are initiated and sustained by factors produced or released by the subgingival microflora.[9] Some of these substances can directly damage host cells and tissues, while others activate endogenous cellular and humoral inflammatory systems which secondarily affect the integrity of the periodontium. Several studies have discussed the role of NO in the progression of periodontal diseases in human beings. Chen *et al*. revealed an increase in NO expression in periodontitis whereas Lohianai *et al*.[10] demonstrated that enhanced formation of NO played a significant role in the pathogenesis of periodontitis. Kankanian *et al.,* on the other hand, opined that the stimulation of NO synthesis was a possible protective function of the saliva and its disorders in periodontal diseases. Akopov and Kankanian[11] assumed the deactivation of NO by activated PMNs as one of the pathomechanisms of disorders in periodontitis. Thus, the present status of the role of NO in periodontal disease is not clearly defined and this study was therefore undertaken to reveal the involvement of NO in periodontal disease.

The results of our study showed significantly increased concentrations of nitrite in patients with periodontitis, as compared to the healthy control group. This finding was expected as it was shown that nitric oxide synthesis is increased in inflamed periodontal tissues. Also, studies have shown increased salivary concentrations of nitric oxide in patients with periodontitis as compared to healthy individuals.[12] Another study concluded that oral *de novo* nitric oxide production increases during deposition of dental plaque which might be an early host defence mechanism against bacterial proliferation in plaque.

The significantly higher levels of NO in the case group may contribute to the development of the frequently found clinical symptoms of periodontitis. Gingival redness may be explained by the vasodilatory effect of NO, and the gingival swelling by the vascular permeability-increasing effect of NO. The increased tendency of the soft tissue to bleed on gentle probing may be due to the inhibitory effect of NO on platelet aggregation and the adhesion-inhibitory effect of NO.[10] The increased alveolar bone resorption may be due to the stimulatory effect of NO on the activity of the osteoclasts. Besides the cytotoxic and tissue-damaging effect of NO itself, increased prostaglandin E_2_ production due to the stimulating effect of NO on COX activity, has been shown to play a synergistic role in osteoclastic bone resorption and vasodilation.[13]

Periodontal diseases are chronic inflammatory infections associated with Gram-negative bacteria, including *Porphyromonas gingivalis*, *Prevotella intermedia*, and *Actinobacillus actinomycetemcomitans*, which stimulate nitric oxide (NO) production.[14] Moreover, NO is increased in inflamed gingival tissue;[15] expression of various cytokines and inducible nitric oxide (iNOS) may be involved in the inflammatory process in periodontitis. The correlation between increased NO levels in gingival fluid and decreased colony-forming units (CFU) of the periodontal pathogen, *P. intermedia,* in deep pockets (> 6 mm), suggested a possible microbicidal effect[16] of NO. Nitric oxide induced by iNOS has been shown to possess immunomodulatory, cytotoxic, and antibacterial effects,[17] consistent with a role for reactive oxygen and nitrogen species in periodontal tissue damage as well as in microbial killing. Studies have shown that rapid serum diffusion of NO could contribute to increased aqueous nitrite and nitrate levels, implicating NO in the pathophysiology and progression of diabetic retinopathy, as well as in periodontal disease.[18]

Recently it has been proposed that selective inhibition may be a promising novel approach for the treatment of periodontitis. Mercaptoethylguanidine (MEG), a selective inhibitor of iNOS and a scavenger of peroxynitrite,[19] was shown to significantly reduce plasma extravasation in gingivomucosal tissue, and decrease the degree of alveolar bone destruction in a rat model of periodontitis. Along the same lines, subgingival local delivery of NO inhibitors might be useful in the treatment of periodontal inflammation.

Further studies must be undertaken to assess nitrite levels in other forms of periodontitis and gingivitis and compared within subgroups to reveal expression of NO during different stages of periodontal disease progression.

## CONCLUSION

Analyzing our data, we come to the conclusion that low concentrations of basally produced nitric oxide maintain normal homeostasis, and are protective under physiological conditions in circumdental tissues. However, NO may be detrimental when produced in excess in inflammation, and may destroy the host tissues and the invading microorganisms. Thus, Nitric oxide concentration in serum can be used as an inflammatory marker for disease status and progression.

## References

[1] Lohinai ZM, Szabo C (1998). Role of nitric oxide in physiology and pathophysiology of periodontal disease. Med Sci Monit.

[2] Stefenovic-Racic M, Stadler J, Evans CH (1993). Nitric oxide and arthritis. Arthritis Rheum.

[3] Michael T, Feron O (1997). Nitric oxide synthesis: Which, where, how and why?. J Clin Invest.

[4] Kwon NS, Nathan CF, Gilker C, Griffith OW, Matthews DE, Stuehr DJ (1990). L-citrulline production from L-arginine by macrophage nitric oxide synthase. The ureido oxygen derives from dioxygen. J Biol Chem.

[5] Forstermann U, Schmidt HH, Pollock JS (1991). Isoforms of NOS, characterization and purification from different cell types. Biochem Pharmocol.

[6] Offenbacher WA (1990). Biochemical mediators of the host response in periodontal diseases. J Dent Res.

[7] Palmer RMJ, Ferrige AG, Moncadab S (1987). Nitric oxide release accounts for the biological activity of endothelium derived relaxing factor. Nature.

[8] Bennett BM, Kobus SM, Brien JF, Nakatsu K, Marks GS (1986). Requirement for reduced unligandedhaemoprotein for heamoglobin and myoglobin mediated biotransformation of glyceryltrinitrate. J Pharmacol Exp Ther.

[9] Kahnberg KE, Lindhe J, Hellden L (1976). Initial gingivitis induced by topical application of plaque extract. A histomertric study in dogs with normal gingival. J Periodontal Res.

[10] Lohinai Z, Benedek P, Fehér E, Györfi A, Rosivall L, Fazekas A (1998). Protective effects of mercaptoethylgnanidine, a selective inhibitor of inducible nitric synthase, on ligature-induced periodontitis in the rat. Br J Pharmacol.

[11] Akopov SE, Kankanian AP (1996). Nitric oxide inactivation by polymorphoneuclear leucocytes as mechanism for the development of periodontal lesions. Stomatologiia (Mosk).

[12] Matejka M, Partyka L, Ulm C, Solar P, Sinzinger H (1998). Nitric oxide synthesis is increased in periodontal disease. J Periodontal Res.

[13] Inoue T, Fukuo K, Morimoto S, Koh E, Ogihara T (1993). Nitric oxide mediates interleukin-1 induced prostaglandin E_2_ production by vascular smooth muscle cells. Biochem Biophys Res Commun.

[14] Kim SJ, Ha MS, Choi EY, Choi JI, Choi IS (2004). Prevotella intermedia lipopolysaccharide stimulates release of nitric oxide by inducing expression of inducible nitric oxide synthase. J Periodontal Res.

[15] Hirose M, Ishihara K, Saito A, Nakagawa T, Yamada S, Okuda K (2001). Expression of cytokines and inducible nitric oxide synthase in inflamed gingival tissue. J Periodontol.

[16] Skaleric U, Gaspirc B, McCartney-Francis N, Masera A, Wahl SM (2006). Proinflammatory and Antimicrobial Nitric oxide in gingival fluid of Diabetic patients with Periodontal disease. Infect Immun.

[17] Zamora R, Vodovotz Y, Billiar TR (2000). Inducible nitric oxide synthase and inflammatory disease. Mol Med.

[18] Tsai DC, Chiou SH, Lee FL, Chou CK, Chen SJ, Peng CH (2003). Possible involvement of nitric oxide in the progression of diabetic retinopathy. Opthalmologica.

[19] Szabó C, Ferrer-Sueta G, Zingarelli B, Southan GJ, Salzman AL, Radi R (1997). Mercaptoethyguanidine and guanidine inhibitors of nitric oxide synthase react with peroxinitrite and protect against peroxinitrite-induced oxidative damage. J Biol Chem.

